# 

**Published:** 1894-06

**Authors:** 


### CONTENTS FOR JUNE, 1894.

ORIGINAL COMMUNICATIONS. PAGE.

The Cause of Typhoid Fever in General, and the Cause of the Epidemic in Buffalo during March in Particular. By CHARLES CARY, M. D. .... 641
The Prevention of Disease—A Problem for all Physicians. By WILLIAM WARREN Potter, M. D. .... 647
Maritime Quarantine. By WILLIAM A. WHEELER, M. D. .... 659

SELECTIONS.

Establishing a New Method of Artificial Respiration in Asphyxia Neonatorum. By J. HARVIE DEW, with illustrations .... 665
Abdominal Surgery in North Germany .... 673

EDITORIAL.

College Commencements and Alumni Association Meetings .... 679
Topics of the Month .... 687
Personal .... 691
Obituary .... 693
Society Meetings .... 693
Hospital Notes .... 694
Academy of Medicine Notes .... 695
Book Reviews .... 695
Literary Notes .... 703

